# Bridge technique for hemifacial spasm with vertebral artery involvement

**DOI:** 10.1007/s00701-021-05006-8

**Published:** 2021-10-06

**Authors:** Takuro Inoue, Satoshi Shitara, Yukihiro Goto, Abrar Arham, Mustaqim Prasetya, Lori Radcliffe, Takanori Fukushima

**Affiliations:** 1 Department of Neurosurgery, Subarukai Koto Memorial Hospital, 2-1 Hiramatsu-cho, Higashiohmi-shi, Shiga 527-0134 Japan; 2 Department of Neurosurgery, Indonesia National Brain Center Hospital, East Jakarta, Special Capital Region of Jakarta Indonesia; 3 Raleigh Neurosurgical Clinic, Raleigh, NC USA; 4 Division of Neurosurgery, Duke University Medical Center, Durham, NC USA

**Keywords:** Bridge technique, Hemifacial spasm, Microvascular decompression, Supraolivary fossette, Surgical technique, Vertebral artery

## Abstract

**Background:**

To assess efficacy and safety of a newly developed decompression technique in microvascular decompression for hemifacial spasm (HFS) with vertebral artery (VA) involvement.

**Methods:**

A rigid Teflon (Bard^®^ PTFE Felt Pledget, USA) with the ends placed between the lower pons and the flocculus creates a free space over the root exit zone (REZ) of the facial nerve (bridge technique). The bridge technique and the conventional sling technique for VA-related neurovascular compression were compared retrospectively in 60 patients. Elapsed time for decompression, number of Teflon pieces used during the procedure, and incidences of intraoperative manipulation to the lower cranial nerves were investigated. Postoperative outcomes and complications were retrospectively compared in both techniques.

**Results:**

The time from recognition of the REZ to completion of the decompression maneuvers was significantly shorter, and fewer Teflon pieces were required in the bridge technique than in the sling technique. Lower cranial nerve manipulations were performed less in the bridge technique. Although statistical analyses revealed no significant differences in surgical outcomes except spasm-free at postoperative 1 month, the bridge technique is confirmed to provide spasm-free outcomes in the long-term without notable complications.

**Conclusions:**

The bridge technique is a safe and effective decompression method for VA-involved HFS.

## Introduction

Involvement of the vertebral artery (VA) in neurovascular compression (NVC) is not a rare condition in microvascular decompression (MVD) for hemifacial spasm (HFS) [15, 18, 19]. The VA can be a sole offender unilaterally or bilaterally [15]. Concomitant small arteries, such as the anterior inferior cerebellar artery (AICA) and the posterior inferior cerebellar artery (PICA), are frequently observed in VA involvement [7, 18, 21]. VA-involved HFS is technically challenging and complex due to the large diameter and toughness of the VA in a narrow operative field. Manipulation at the vicinity of the vital area carries risks of injury to the cranial nerves and the brainstem perforators [6, 12, 20]. Many decompression techniques were reported in relation to VA-involved HFS. Some of them are not always feasibly performed and require extensive manipulation of the lower cranial nerves [1–5, 7, 9–11, 13, 18, 22]. A variety of decompression methods for individual anatomical differences contribute to the safety of MVD surgery. We introduce our simple decompression technique for VA-involved HFS and assessed its efficacy and sustainability in the long-term in comparison with the conventional sling technique.

## Methods

### Patient cohort

**Fig. 1:**
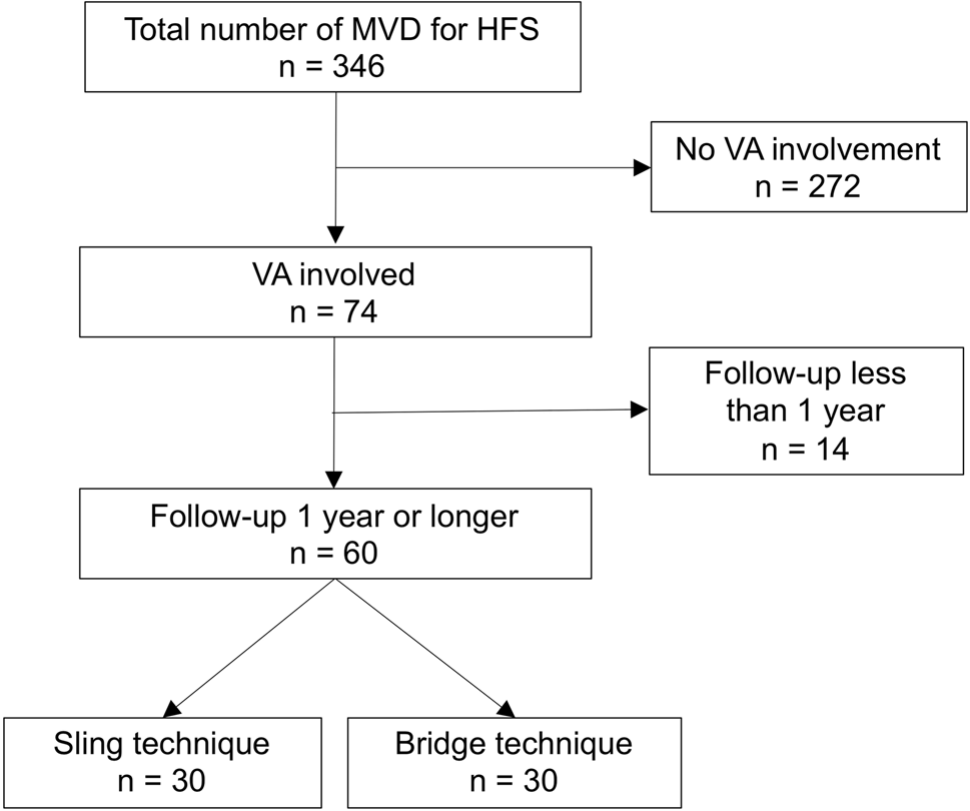
Participant flow diagram. MVD, microvascular decompression; HFS, hemifacial spasm; VA, vertebral artery

VA involvement was identified in 74 patients (21%) among 346 patients with HFS treated in our institutes from July 2004 to June 2020. Patients with a follow-up period of less than 1 year were excluded from this study. We retrospectively compared 60 patients who underwent MVD using two different transposition techniques. The bridge technique was used in 30 patients and the conventional sling technique (relocation with a Teflon sling and a small wedge insertion if required) was applied in the remaining 30 patients (Fig. 1).

### Operative technique

**Fig. 2:**
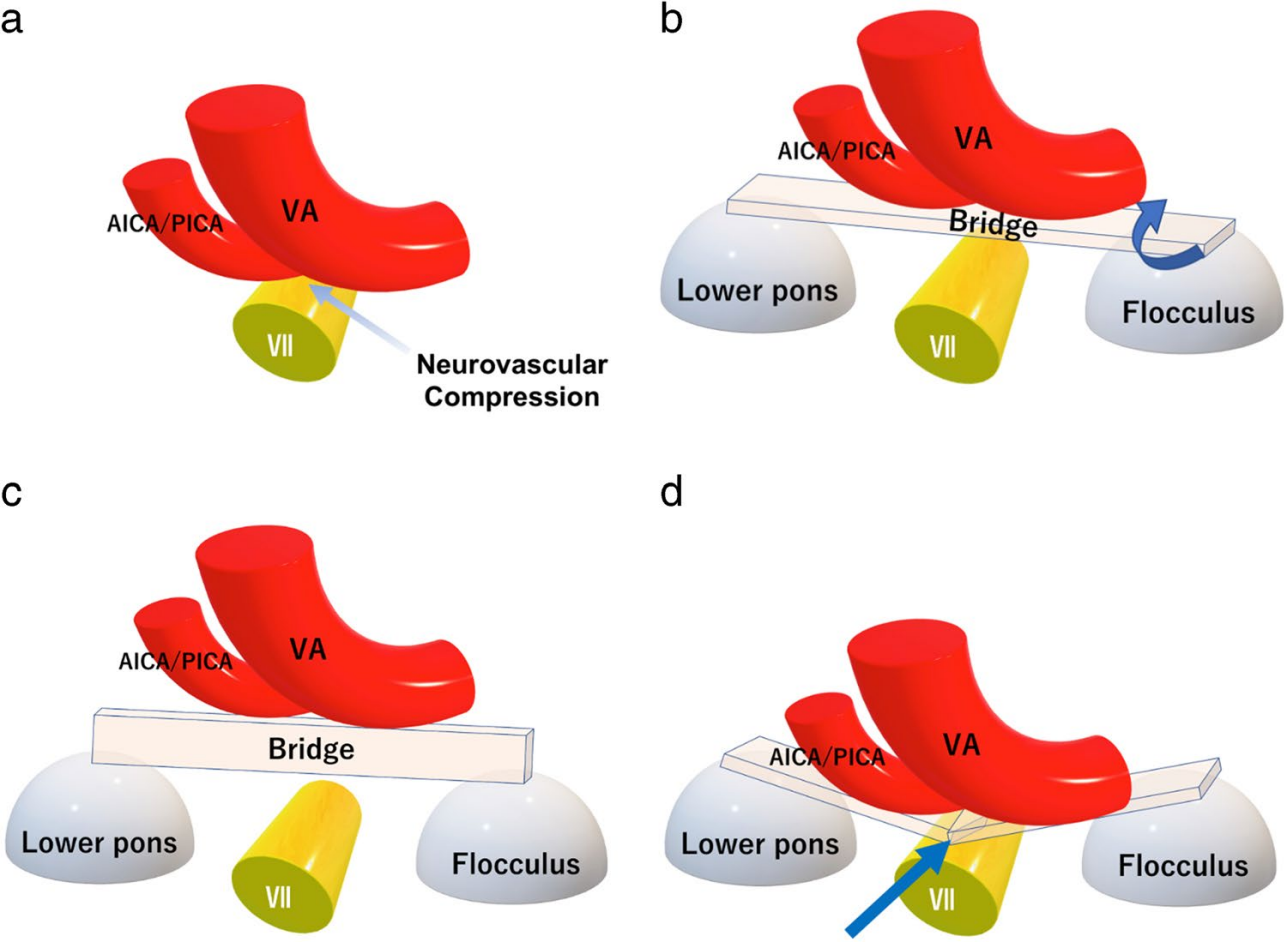
Schematic illustration of the bridge technique. **a** A schematic illustration of the vertebral artery (VA)-involved neurovascular compression (NVC). The anterior inferior cerebellar artery (AICA) and/or the posterior inferior cerebellar artery (PICA) are concomitantly involved in most cases. **b**, **c** The NVC is located at the supraolivary fossette (SOF), which is deeper than the lower pons and the flocculus. A Teflon bridge is first inserted parallel with the brainstem surface, then rotated 90 degrees (blue curved arrow) to reinforce holding the offenders and create free space on the root entry zone (REZ). The edges of the bridge are placed on the brainstem and the flocculus. **d** A schematic figure of a failed transposition. When a bridge is not rotated, the bridge may bend by compression force from the VA, resulting in re-compression onto the nerve root (blue arrow)

**Fig. 3:**
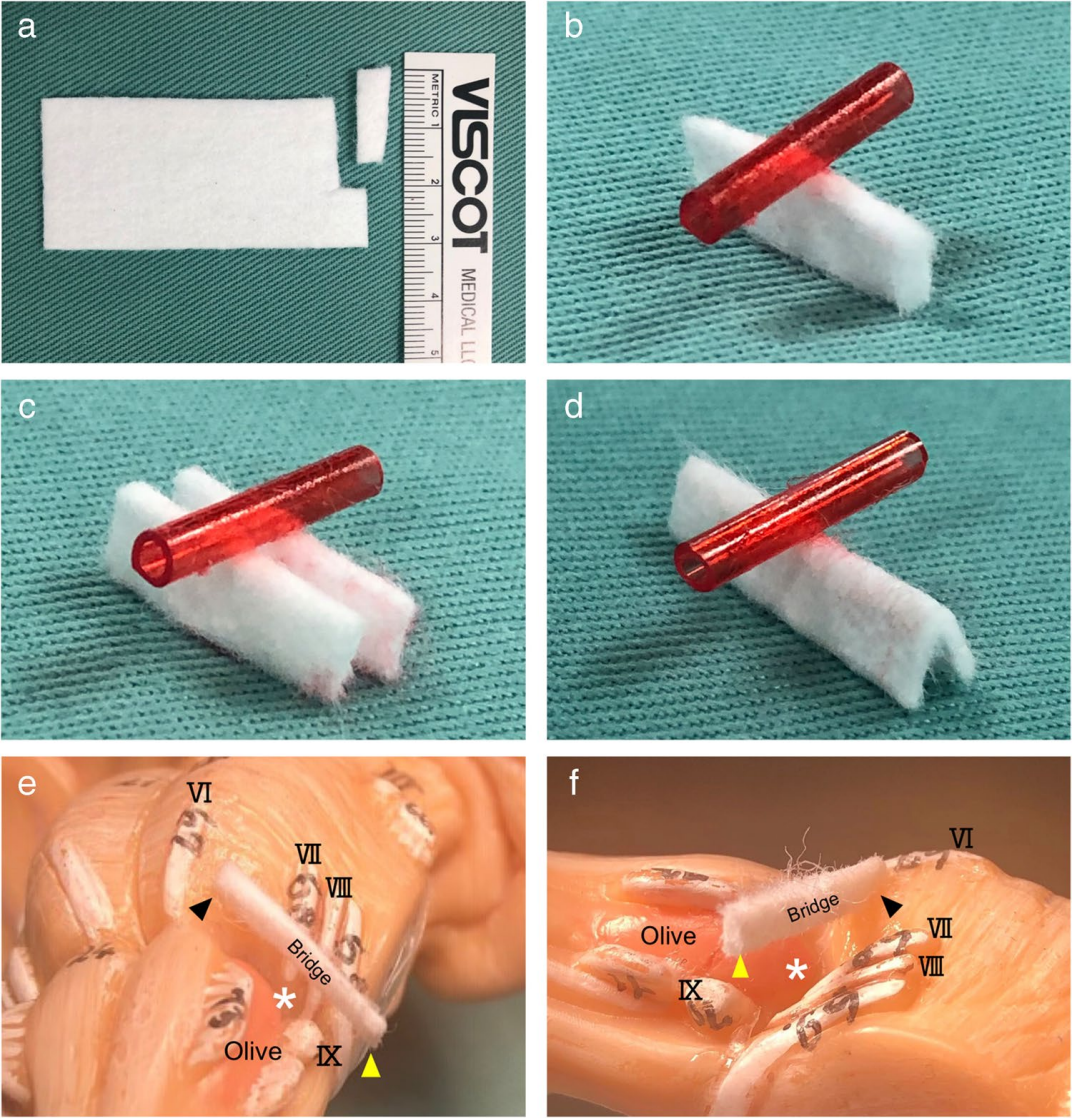
Figure presentation of the bridge technique. **a** A Teflon bridge cut out from a Teflon sheet (Bard^®^ PTFE Felt Pledget, nominal thickness 1.65 mm). Appropriate length of 15–18 mm and width of 3–4 mm. **b** A schematic figure elevating the VA (red tube) with a single Teflon bridge. **c** A dual bridge for high-compression force from the VA. One of the bridges can be placed either above or below the lower cranial nerves. **d** A folded bridge is another method for high-compression force from the VA. A longitudinally folded Teflon bridge can reinforce supporting the VA. **e** A brainstem figure with a Teflon bridge shows the typical placement of a bridge over the SOF. The medial edge is placed on the brainstem near the abducens nerve (black arrowhead). The lateral edge is placed on the bottom of the flocculus (yellow arrowhead). A small free space is created over the REZ of the facial nerve (white asterisk). **f** The same figure looking from the lateral shows a free space (white asterisk) created over the REZ on the SOF

All microvascular decompression procedures were performed by the first author. Patients were operated through the retrosigmoid approach in the lateral position with monitoring of auditory brainstem evoked response and lateral spread response. The cerebellar hemisphere is gently elevated from the base of the posterior fossa, and then, the lower cranial nerves were identified. After retracting the flocculus and the choroid plexus, the root entry zone (REZ) of the facial nerve was identified at the superior aspect of the supraolivary fossette. The NVC was confirmed to be at the facial nerve by using a facial nerve stimulator. The offending vessels were elevated using a suction tube or a dissector to obtain sufficient space over the REZ. This manipulation was performed mainly between the glossopharyngeal nerve and the cochlear-vestibular nerve. The proximal side of the VA is kept untouched to avoid lower cranial nerve injury except in the cases with a tough VA, in which case the VA is manipulated through or below the lower cranial nerves. The concept of the bridge technique is shown in Fig. 2. A rectangular Teflon board inserted parallel with the brainstem surface over the REZ (Fig. 2a, b) is rotated 90 degrees to hold the offenders securely (Fig. 2c) to prevent collapse by the vascular compression force (Fig. 2d). A single Teflon bridge, which is cut out from a Teflon sheet (Fig. 3a, Bard^®^ PTFE Felt Pledget, nominal thickness 1.65 mm), approximately 15–18 mm in length and 3–4 mm in width, is placed vertical to the axis of the VA to maintain the elevated position (Fig. 3b). The medial end of the bridge is placed on the lower pons near the abducens nerve root exit. The lateral end is on the base of the flocculus to create free space over the REZ (Fig. 3e, f). In cases with a tough VA, either a dual bridge (Fig. 3c) or a folded bridge (Fig. 3d) is used depending on the compression force of the VA. No fibrin glue is used in this technique. For the sling technique, multiple slings made from shredded Teflon felt are wrapped around each offender; then, reposition is carried out by attaching the slings to the petrous dural surface. Fibrin glue is applied to fixate the slings onto the petrous dura. Supportive small Teflon wedges and/or balls are used to secure the transposition in both techniques, if required. In both techniques, no Teflon prosthesis is placed directly on the REZ. Elapsed time from recognition of the REZ to completing the decompression procedure (decompression time), the number of inserted Teflon felts (No. of Teflon pieces), and incidences of manipulating the proximal side of the VA through or below the lower cranial nerves (LCN manipulation) were investigated by viewing the operative video records.

### Assessment of outcomes

The postoperative neurological status of the patients was assessed either at our clinic, by telephone interviews, or by mailed questionnaires for remote patients. The status of the facial spasm was evaluated at postoperative 1 week, 1 month, and 1 year. Any neurological complications, including post-operative facial palsy, delayed facial palsy, hearing impairment, hoarseness, dysphagia, cerebrospinal fluid leakage, and infectious complications, were recorded. Computed tomography (CT) was performed in all patients postoperatively. Magnetic resonance imaging (MRI) was performed to evaluate possible migration of the Teflon bridge later than 1 month postoperatively.

In describing demographic characteristics, *P* values across dura closure categories were obtained by linear regression for continuous variables and by an *χ*^2^ test or the Mantel–Haenszel test for categorical variables. Each statistical test was set to be significant at *P* < 0.05 (2-sided *P* value). The SAS software (version 9.4; SAS Institute, Inc., Cary, North Carolina) was used for all statistical analyses.

## Results

Patient characteristics, operative outcomes, and follow-up results are summarized in Table 1. The mean age at MVD was 55 years. No sex predominancy was found. The median duration of symptoms before MVD was 4 years in total. The left side was predominantly affected more than the right side (left, 87%; right, 13%). Patient characteristics were not significantly different between the two techniques (Table 1).

**Table 1:** Summary of patient characteristics, operative outcomes, and follow-up results

Patient characteristics	All	Sling (*n* = 30)	Bridge (*n* = 30)	*P* value
Mean age at MVD, years (range)	55 (31–84)	58 (33–76)	52 (31–84)	0.0647
Sex (male/female)	30 (50%)/30 (50%)	13 (43%)/17 (57%)	17 (57%)/13 (43%)	0.3058
Affected side (right/left)	8 (13%)/52 (87%)	6 (20%)/24 (80%)	2 (7%)/28 (93%)	0.132
Median/mean symptom duration before MVD, years (range)	4/4.6 (0.5–20)	3/4 (0.5–20)	5/5 (0.5–12)	0.2828
Number of redo cases	4 (6.7%)	1 (3.3%)	3 (10%)	0.3047
Operative outcomes and follow-up				
Median/mean follow-up, months (range)	46/55(13–126)	73/75 (16–126)	36/34 (13–47)	< .0001*
Spasm-free after MVD				
1 week	55 (92%)	25 (83%)	30 (100%)	0.0522
1 month	54 (90%)	24 (80%)	30 (100%)	0.0237*
1 year	52 (87%)	23 (77%)	29 (97%)	0.0523
Last follow-up	54 (90%)	25 (83%)	29 (97%)	0.1945
Failure	3 (5%)	3 (10%)	0 (0%)	0.2373
Recurrence	3 (5%)	2 (6.7%)	1 (3.3%)	1
Complications				
Delayed facial palsy	6 (10%)	4 (13%)	2 (6.7%)	0.6707
Neurological complications (transient/persistent)				
Facial weakness	3/0 (5%/0%)	3/0 (10%/0%)	0/0 (0%/0%)	NA
Hearing impairment	2/2 (3.3%/3.3%)	2/2 (6.7%/6.7%)	0/0 (0%/0%)	NA
Hoarseness	2/1 (3.3%/1.7%)	1/0 (3.3%/0%)	1/1 (3.3%/3.3%)	NA
Swallowing disturbance	1/0 (1.7%/0%)	0/0 (0%/0%)	1/0 (3.3%/0%)	NA
Other complications				
CSF leak	0 (0%)	0 (0%)	0 (0%)	NA
Meningitis	1 (1.7%)	0 (0%)	1 (3.3%)	NA
Wound infection	1 (1.7%)	0 (0%)	1 (3.3%)	NA

*MVD* microvascular decompression, *CSF* cerebrospinal fluid; *, significant difference; *NA*, not available

**Fig. 4:**
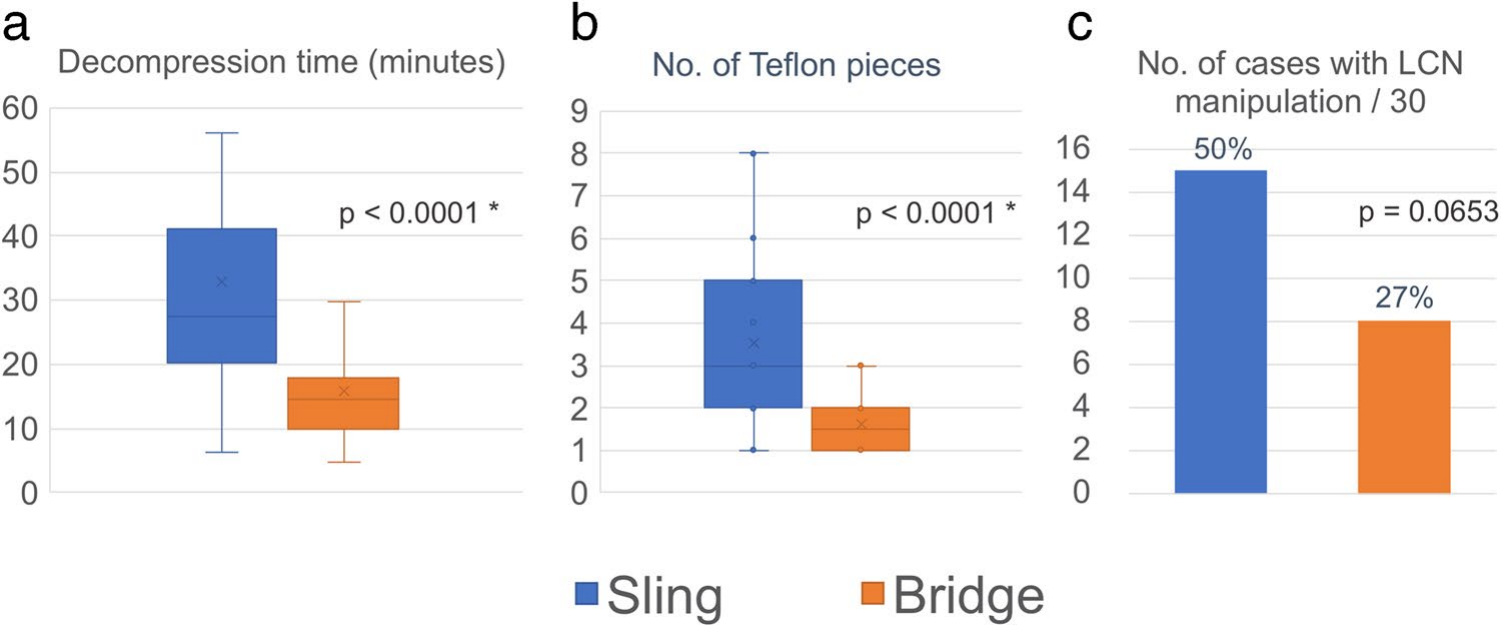
Data analyses of operative manipulations. **a** Decompression time (blue column, sling technique; orange column, bridge technique) from identification of the REZ to completing the decompressive maneuvers in a single procedure. (*, significant difference) **b** Number of Teflon pieces used for decompression in a single MVD. **c** Number of cases requiring lower cranial nerve manipulation among 30 patients in each technique. LCN, lower cranial nerves

Intraoperative findings revealed that the AICA and/or the PICA were concomitantly involved with the VA as offenders in most cases (AICA, 72%; PICA, 53%, in total). Comparison of surgical data between the two techniques is shown in Fig. 4. Decompression time was significantly shorter in the bridge technique (mean 16 min) than in the sling technique (mean 33 min). Fewer Teflon pieces were used in the bridge technique than in the sling technique, the mean of 1.6 pieces versus 3.5 pieces, respectively, which was significantly different in statistical analysis. LCN manipulation was performed less in the bridge technique in 8 patients (27%) than in the sling technique in 15 patients (50%) (Fig. 4).

The follow-up period was significantly longer in the sling technique than in the bridge technique because the bridge technique commenced later (Table 1). Spasm-free was obtained in all patients (100%) in the bridge technique, but only 25 patients (83%) in the sling technique, 1 week postoperatively. The spasm-free rate was 100% and 97% in the bridge technique, but 80% and 77% in the sling technique at postoperative 1 month and 1 year, respectively. During the entire follow-up period, 29 patients (97%) in the bridge technique and 25 patients (83%) in the sling technique became spasm-free. No patient with decompression failure was observed in the bridge technique, while 3 patients (10%) were counted as decompression failure in the sling technique. Recurrence of facial spasm was observed in one patient (3.3%) in the bridge technique and 2 patients (6.7%) in the sling technique, respectively. Despite the bridge technique demonstrating better outcomes in regard to efficacy, statistical analyses revealed no significant differences except spasm-free at postoperative 1 month (Table 1).

**Fig. 5:**
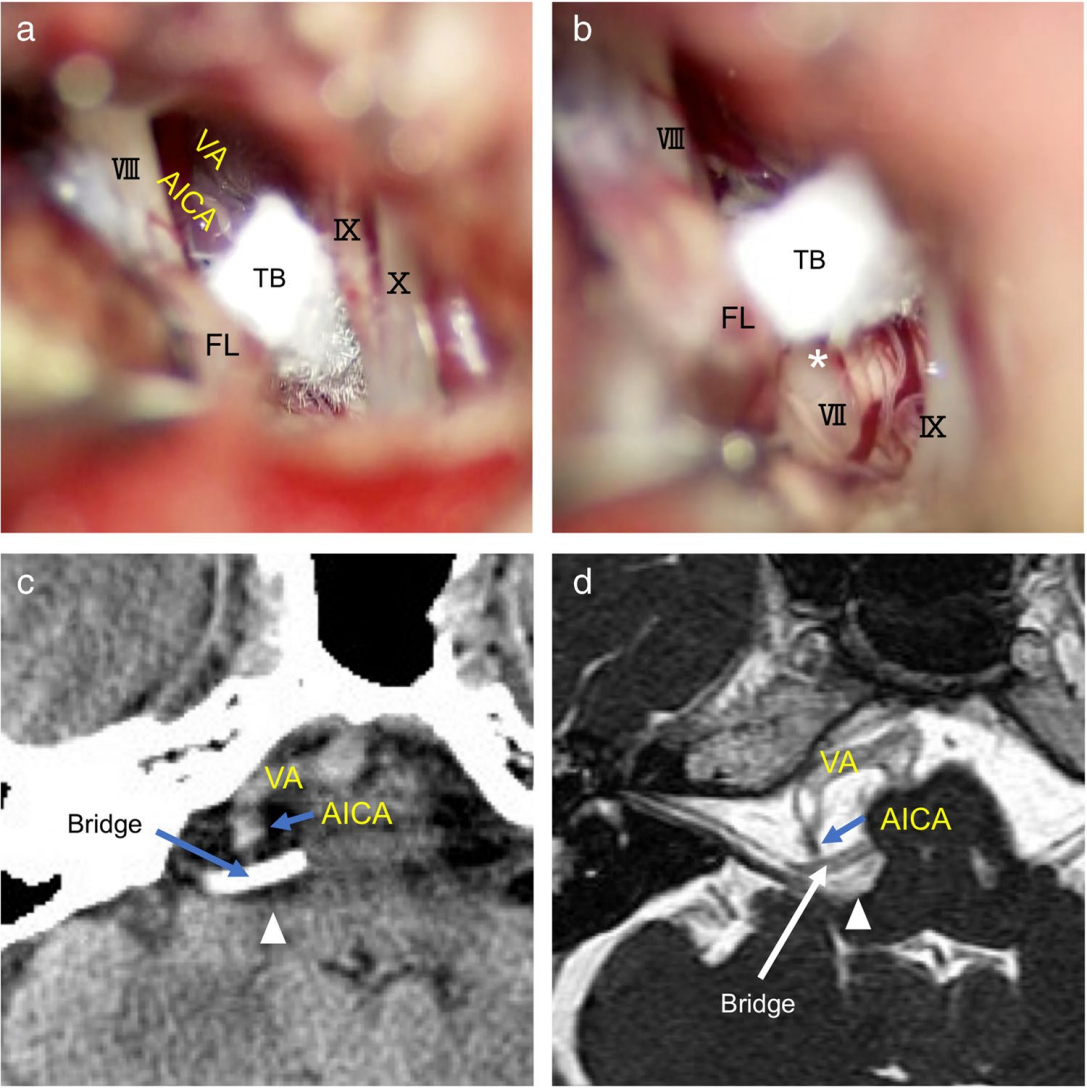
Case presentation (Case 1). A 70-year-old woman with HFS on the right caused by the VA and AICA compression. **a** An intraoperative photograph of MVD for hemifacial spasm on the right side. A Teflon bridge (TB) is inserted to reposition the offenders, the VA, and the AICA. The lateral edge is placed on the base of the flocculus (FL), and the other edge is placed on the brainstem near the exit of the abducens nerve. **b** A photo from the caudal side shows a free space over the root exit zone (REZ) of the facial nerve (white asterisk) created under the bridge. **c** A postoperative computed tomography (CT) taken postoperative1 week indicates the location of the bridge (white rectangular shape) and a free space over the REZ (white arrowhead). **d** Magnetic resonance imaging (MRI) taken 6 months after surgery shows a free space (white arrowhead) maintained in front of the REZ and the transposed offenders in place on the Teflon bridge

**Fig. 6:**
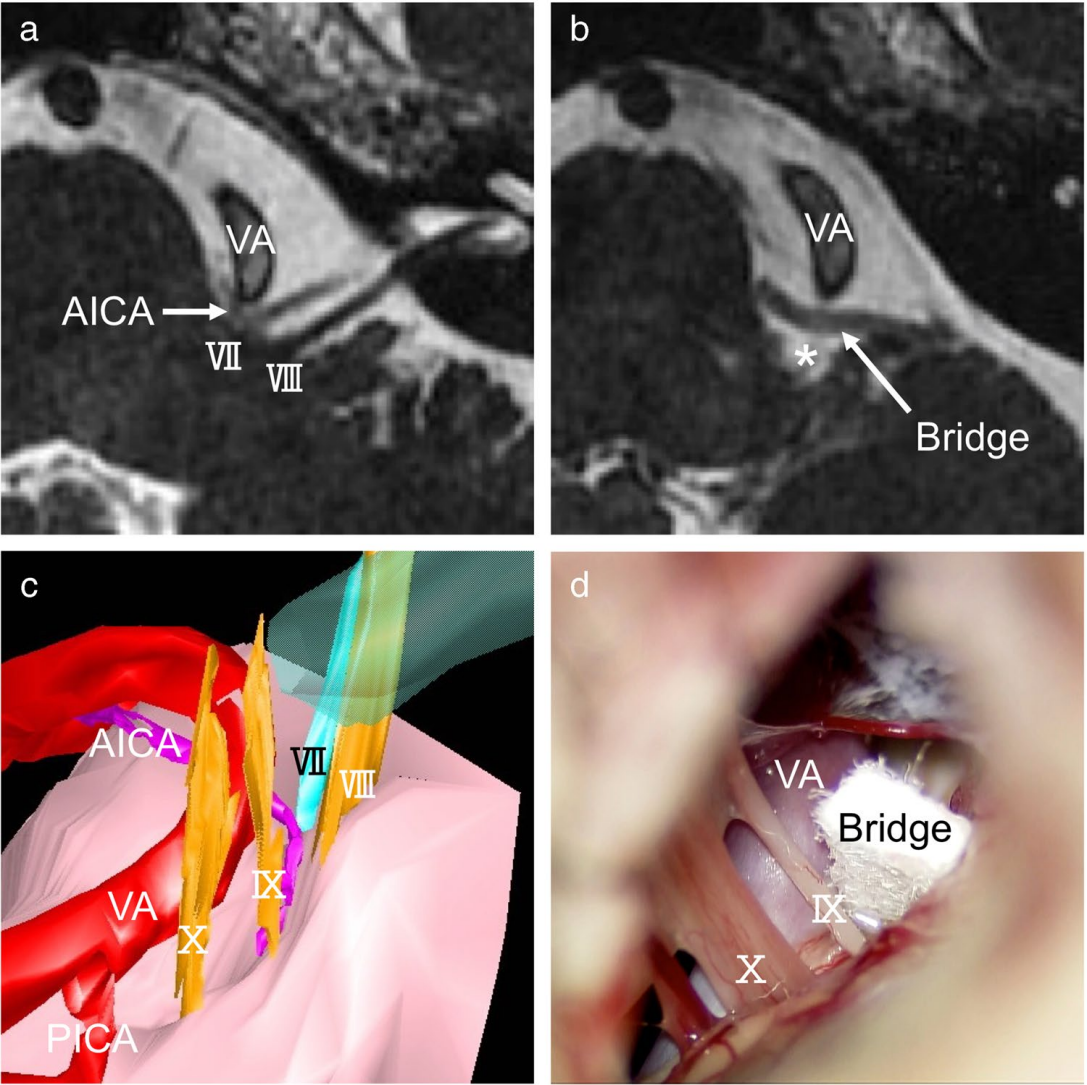
Case presentation (Case 2). A 54-year-old man with HFS on the left due to the VA and the AICA. **a** An MRI slice shows neurovascular compression by the AICA in combination with the VA on the facial nerve on the left side. **b** An MRI taken 1 year after MVD demonstrates the inserted bridge in place and a free space maintained over the REZ (asterisk). **c** A pre-operative three-dimensional (3D) image shows a relationship between the facial nerve and the adjacent structures. The AICA is the direct compression onto the REZ. The VA should be repositioned simultaneously to obtain sufficient decompression. **d** An intraoperative photograph indicating a single Teflon bridge inserted just above the ninth cranial nerve root to reposition the offenders, the VA, and the AICA. The lateral edge is placed on the flocculus, and the other edge is placed on the brainstem near the exit of the abducens nerve

Delayed facial palsy was noted in 2 patients (6.7%) in the bridge technique and in 4 patients (13%) in the sling technique. Postoperative immediate facial weakness and hearing impairment were observed only in the sling technique, 3 and 2 patients respectively. Facial weakness was transient in all patients; however, hearing impairment became permanent in all patients in the sling technique. Lower cranial nerve injury, such as hoarseness and swallowing disturbance, was noted in one patient in each technique. Hoarseness in the bridge technique did not recover. During surgery for this patient, the lower cranial nerves were widely split, and two Teflon bridges were inserted through and above the lower cranial nerves. Wound infection and meningitis were observed in one patient in each technique. Statistical analysis was not performed due to a small number of complications in each group (Table 1). No migration of the inserted Teflon bridges was observed in any patients on the follow-up CT and MRI (Figs. 5c, d, 6b).

### Case presentations

#### Case 1 (Fig. 5)

A 70-year-old woman suffered from HFS on the right for 2 years. Medication was not effective to reduce her deteriorating symptom and an MVD was considered. MRI revealed the AICA in combination with the VA were the offenders. A single Teflon bridge was inserted between the flocculus and the brainstem to elevate the offenders (Fig. 5a). A free space over the REZ was confirmed intraoperatively (Fig. 5b). Her facial spasm disappeared immediately after surgery, and she maintained spasm-free for more than 1 year of follow-up. A CT scan taken postoperative 1 week (Fig. 5c) and an MRI taken postoperative 6 months (Fig. 5d) showed the free space over the REZ was maintained.

#### Case 2 (Fig. 6)

A 54-year-old man with HFS on the left was treated with MVD. Medication and Botulinus toxin injection failed to improve his symptom. The offenders were the AICA and the VA (Fig. 6a). A pre-operative three-dimensional (3D) image indicated transposition required both the AICA and the VA to obtain a sufficient nerve decompression (Fig. 6c). A Teflon bridge was used to elevate the offenders just above the glossopharyngeal nerve root (Fig. 6d). The patient became spasm-free immediately after surgery and maintained spasm-free at the last follow-up of 1 year. An MRI postoperative 1 year confirmed a free space maintained over the REZ (Fig. 6b).

#### Case 3 (Fig. 7)

**Fig. 7:**
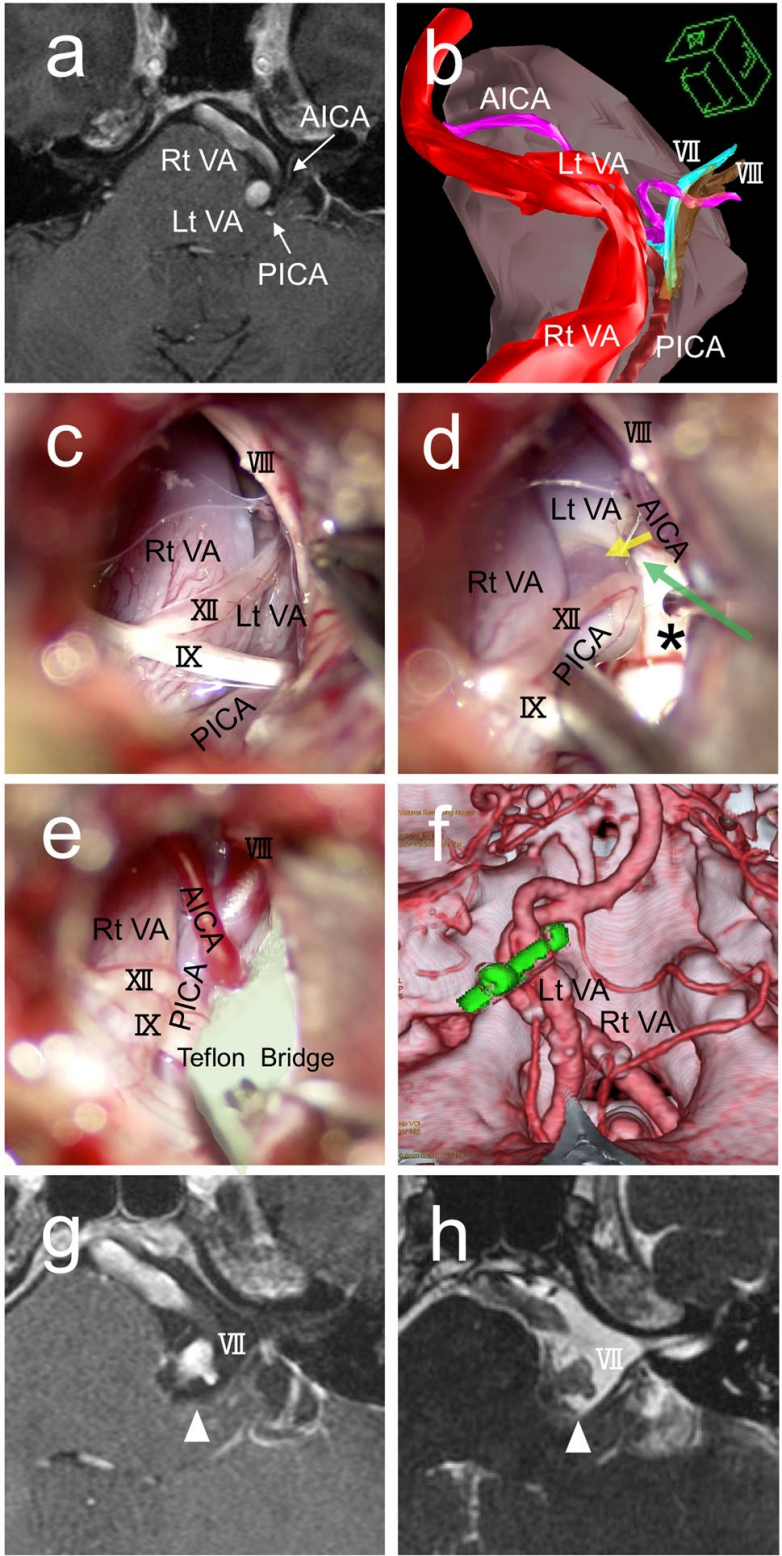
Case presentation (Case 3). A 44-year-old man with HFS on the left. The VAs on both sides, the PICA, and the AICA are involved. **a** A Gadolinium-enhanced MRI shows multiple offenders present near the REZ of the facial nerve on the left side. **b** A three-dimensional (3D) image looking from the lower-left clarifies the anatomical relationships of the offending arteries and the facial and vestibular nerves. **c** An intra-operative photograph shows the cistern is occupied with the offenders and the stretched cranial nerves. The hypoglossal nerve (XII) is at an unusual location stretched by the PICA orifice from the left VA. **d** A photo showing identification of the REZ of the facial nerve (black asterisk) after elevating both vertebral arteries and the PICA away from the brainstem. The AICA is still deeply impinging onto the REZ of the facial nerve. The AICA is relocated laterally (yellow arrow) together with other offenders, and then, a Teflon bridge is inserted into the gap (green arrow). **e** All offenders are held with a Teflon bridge (colored in light green). Two additional small Teflon pieces are placed on both edges to secure the position of the bridge. **f** A post-operative 3D-CT demonstrates the bridge (green) is holding all offenders. **g** An enhanced MRI shows the offenders elevated away from the facial nerve with a free space over the REZ (white arrowhead). **h** An MRI with fast imaging employing steady-state acquisition shows cerebrospinal intensity over the REZ of the facial nerve (white arrowhead)

A 44-year-old man suffered from severe hemifacial spasm on the left for 5 years. He underwent his first MVD elsewhere, resulting in failure due to a complex neurovascular compression. Two years later, he was evaluated with 3D images on MRI at our institute (Fig. 7a, b). The direct compression on the REZ was from the AICA, accompanied by the PICA orifice from the left VA. The bilateral VAs were occupying the cistern and hampering the manipulation to the REZ (Fig. 7c). To achieve nerve decompression, all four vessels needed to be relocated. A folded Teflon bridge was used to elevate all offenders (Fig. 7d, e). Supportive Teflon pieces were added on both ends to ensure the bridge fixation. A postoperative 3D-CT and MRI confirmed the Teflon bridge was in place (Fig. 7f) with a free space created over the REZ of the facial nerve (Fig. 7g, h). The patient became spasm-free immediately after surgery and maintained spasm-free at the last follow-up of 18 months.

## Discussion

A decompression procedure for hemifacial spasm associated with the vertebral artery is more challenging than that for small arteries [6, 12, 18]. Manipulating a tortuous VA in the narrow cistern entails a risk of injury to the adjacent cranial nerves and small perforators. An atherosclerotic and tortuous VA causes not only pure HFS, but also HFS associated with hypertension of neurogenic origin [16, 17]. An ectatic VA is reported to be a factor that relates to the lower success rate and higher incidences of complications [14, 21]. An essential technique to obtain a successful result in cranial nerve compression syndrome is placing a prosthesis with no contact with the nerve root [18]. The sling technique is reported to be a more efficient method than the conventional interposition technique (inserting shredded Teflon pledgets on the REZ) because the REZ is isolated from any contacting object [3–5, 8, 22]. In a narrow operative field with a large VA, however, passing through a Teflon sling around the vessels has potential risks of damaging cranial nerves and perforators. Even if a sling is successfully wrapped around the offender, secure fixation of the tough VA to the petrous dura is another technical difficulty. Some authors reported refined techniques in fixating the VA to the petrous surface, such as using an aneurysm clip, a direct vascular wall suture, or a biomedical glue sling [1–5, 7, 9–11, 22]. Although such methods are possible in cases with a large cistern, a decompression technique should be simpler and safer to avoid accompanying risks [23]. Different from the interposition technique, both the sling and bridge techniques aim to the same surgical achievement, “decompression of the REZ without any prosthesis contact.” The difference is that the sling technique is to “pull-up” the offenders, while the bridge technique is to “push-up” the offenders. The most suitable technique should be applied according to individual variations of NVC. A variety of technical options enable neurosurgeons to manage variable individual anatomies, leading to a successful MVD. One of the risks of MVD is stretching tiny perforators, which may cause ischemic complications of the cranial nerves. The brainstem perforators tend to be stretched in the sling technique because the relocated offenders are fixed away from the REZ to the distant dural surface. An ideal transposition is a secure decompression of the REZ together with minimal stretch of the perforators. Our bridge technique is a unique method that inherits the advantage of the sling technique without its disadvantage. A secure decompression is achieved by dividing the rebound force of the tough VA onto both ends of the bridge by creating an adequate free space over the REZ while avoiding an over-stretch of the perforators. In addition, this free space may even increase as the flocculus returns to the original position postoperatively. To obtain these advantages, a Teflon bridge should be rigid enough not to collapse by compression force from the VA. By rotating a Teflon bridge 90 degrees after insertion parallel with the brainstem surface, the bridge can become tolerant from the compression force and create a secure free space over the REZ. A dual bridge or a folded bridge can be applied to reinforce holding the tougher VA based on the surgeon’s decision during the MVD. An appropriate length bridging between the pontine surface and the flocculus is crucial. The flocculus is soft tissue and the pressure caused by an ectatic VA can be very high, which may result in a collapse of the bridge, becoming merely an interposing of Teflon felt between the artery and the facial nerve. Therefore, the length of the bridge should be long enough to be placed on the flocculus to maintain the compression force, which should measure approximately 15–18 mm in length and 3–4 mm in width according to our experiences.

The concept of the bridge technique was described in the previous literature [13, 18]. Sindou et al. reported a “bridging effect” over the facial nerve by inserting a rectangular Teflon in between the compressive arteries on one side and the lateral aspect of the brainstem and the flocculus on the other side [18]. We noticed the advantages of this technique in our early experiences; however, there was a concern of long-term efficacy due to possible migration of the inserted bridge over time. Our long-term observation confirmed favorable outcomes of this technique and no migration of a bridge, which may be benefited from the characteristics of the material, composed of tiny Teflon fibers on the surface preventing its migration. This study confirmed the usefulness of using a Teflon bridge and demonstrated long-term efficacy and safety for VA transposition. Different from the sling technique, a Teflon bridge can be inserted instantly after elevating all offenders from the REZ. A rotated single bridge may be enough to complete the decompression procedure in most cases when an appropriate size of a bridge is chosen. This study revealed that decompression time is significantly shorter, and fewer numbers of inserted Teflon pieces are required in the bridge technique than in the sling technique, which contributes to the safety of the decompression procedure in MVD. Lower cranial nerve palsy is one of the serious complications of MVD that should be avoided. Manipulation to the proximal side of VA has a potential risk of lower cranial nerve injury [7]. This study demonstrated that the chance of manipulating the lower cranial nerves is reduced in the bridge technique, which may contribute to avoiding these sequelae. Despite the simplicity of the surgical maneuvers, the bridge technique provides superior surgical outcomes in the long-term for patients with VA involvement. Neuronal damage due to placing the Teflon edge onto the lateral aspect of the brainstem was not observed during the observation period in this study. A possible disadvantage of the bridge technique may include adhesion to the glossopharyngeal nerve because the lateral end of a bridge on the flocculus comes close to the glossopharyngeal nerve root. In redo cases with the bridge technique applied, adhesion between the glossopharyngeal nerve and the inserted bridge may disturb the dissection toward the ventro-caudal area of the REZ of the facial nerve. Therefore, in the initial MVD, thorough observation of the entire REZ, using a facial nerve stimulator is crucial. Confirming no remaining offenders left behind the glossopharyngeal nerve is needed before inserting a Teflon bridge.

Limitations of the present study include those that are inherent to studies of retrospective design with a small number of patients and complications. Other limitations include a relatively short follow-up period in the bridge technique and the cases of the surgeon’s early experience of MVD included in the sling technique. A longer period of observation and accumulation of the number of cases are needed to confirm our conclusions.

## Conclusion

The bridge technique for VA-involved hemifacial spasm is comparable to the conventional sling technique in terms of effectiveness and durability in the long-term.
